# Cholecystitis in Pregnancy

**DOI:** 10.1155/S1064744996000592

**Published:** 1996

**Authors:** Brian M. Casey, Susan M. Cox

**Affiliations:** Department of Obstetrics and Gynecology University of Texas Southwestern Medical Center 5323 Harry Hines Boulevard Dallas TX 75235-9032 USA

## Abstract

Biliary tract disease is a relatively uncommon, heterogenous disease in pregnancy. Specifically, acute cholecystitis can be especially difficult to recognize in pregnancy. However, once diagnosed, the initial management plan should be conservative and include antibiotic therapy. Subsequent management depends on the gestational age at diagnosis. Surgical therapy, when indicated, should not be delayed and a planned intervention during the second trimester appears to offer a better outcome than surgery performed under emergent conditions.


Infectious Diseases in Obstetrics and Gynecology 4:303–309 (1996)
© 1997 Wiley-Liss, Inc.

### Cholecystitis in Pregnancy

**Brian M. Casey*** **and Susan M. Cox**
*Department of Obstetrics and Gynecology, University of Texas Southwestern Medical Center, Dallas, Texas*

ABSTRACT

**Biliary tract disease is a relatively uncommon, heterogenous disease in pregnancy. Specifically, acute cholecystitis can be especially difficult to recognize in pregnancy. However, once diagnosed, the initial management plan should be conservative and include antibiotic therapy. Subsequent management depends on the gestational age at diagnosis. Surgical therapy, when indicated, should not be delayed and a planned intervention during the second trimester appears to offer a better outcome than surgery performed under emergent conditions. Infect. Dis. Obstet. Gynecol. 4:303–309, 1996.** © 1997 Wiley-Liss, Inc.

KEY WORDS
biliary tract disease; cholelithiasis; *Escherichia coli; Bacteroides species*

Cholelithiasis is a condition which effects approximately 20 million American women with 1 million new cases diagnosed each year.[1] Women of reproductive age are 4 times more likely than men of similar age to develop gallbladder disease.[2] Epidemiologic studies indicate that the incidence of cholesterol gallstones rises abruptly as women enter the childbearing years.[3] In fact, several studies show a direct relationship between parity and risk of developing biliary stones.[4–6] Generally though, biliary tract disease is a relatively uncommon occurrence during pregnancy. A recent review of more than 45,000 pregnant women revealed the incidence of symptomatic biliary tract disease to be 0.16% in pregnancy.[7] Despite its relatively uncommon occurrence during pregnancy, 81% of women with symptoms of gallbladder disease had their first attack within 1 year of pregnancy. Pregnancy then seems to predispose women to developing biliary tract disease.

Biliary tract disease encompasses a continuum of pathologic entities which includes biliary sludging, cholelithiasis, cholecystitis, choledocholithiasis, and cholangitis. Biliary sludge or the supersaturation of bile with cholesterol, is an important precursor to gallstone formation. The exact prevalence of cholelithiasis in pregnancy is essentially unknown since 50% of women with gallstones are asymptomatic. Efforts to identify asymptomatic cholelithiasis in pregnancy using ultrasound have produced variable results; however, the best estimate of incidence appears to be between 2.5% and 4.5%.[8–10]

In addition to pregnancy, several other factors are strongly associated with gallbladder disease. Ethnicity contributes to the risk for developing biliary disease. In particular, 70% of Native American women older than 30 years of age develop cholelithiasis. Mexican American women have an intermediate prevalence of about 14% with Caucasians and Black women at 4% and 5%, respectively.[11] Chilean women are also reported to be at high risk for developing gallstones.[12] In addition, heredity has been implicated as a contributing factor; 20% of first degree relatives with cholelithiasis will develop symptomatic gallstones.[11] Finally, age and obesity are also commonly associated with biliary disease.

*Correspondence to: Dr. Brian M. Casey, Department of Obstetrics and Gynecology, University of Texas Southwestern Medical Center, 5323 Harry Hines Boulevard, Dallas, TX 75235-9032.

Review Article

Received 10 July 1996

Accepted 1 November 1996

As stated earlier, biliary disease, including acute cholecystitis, occurs infrequently during the pregnant state. In fact, the prevalence of acute cholecystitis in pregnancy ranges from 1/1,000 to 1/10,000 births.[13,14] Cholelithiasis is obviously the major etiologic factor in the development of acute cholecystitis and it has been established that the pregnant state predisposes women to gallstone formation. In light of the key role they play in the etiology of acute cholecystitis, it is only appropriate that a brief description of the pathogenesis of gallstones be provided in this review.

### PATHOGENESIS OF BILIARY STONES

Gallstones are composed of cholesterol, bilirubin, calcium salts, and protein. Each of these ingredients occurs in various percentages. Specifically, there are cholesterol and non-cholesterol stones. Non-cholesterol stones are frequently associated with a chronic hemolytic condition and contain a higher bilirubin concentration. Brown pigment non-cholesterol stones are often associated with infection since bacteria are likely to hydrolyze conjugated bilirubin into calcium salts. This hydrolysis accounts for the brown coloration of many non-cholesterol gallstones.

Cholesterol gallstones, 75% of all stones in the Western world, form when cholesterol concentrations exceed the ability of bile acids to maintain cholesterol in solution. At higher cholesterol concentrations, cholesterol micelles coalesce precipitating crystals. This coalescence yields gallbladder sludge, which is merely thickened mucoprotein with entrapped cholesterol crystals. Sludge is an important precursor to gallstone formation as the crystals bind to the biliary proteins and ultimately result in cholelithiasis.[15]

The increased risk of developing gallstones during pregnancy is due to several factors. First, the gallbladder volume is increased during the second and third trimesters. Second, gallbladder motility is decreased in pregnancy, possibly due to elevated progesterone levels. The evidence, however, is only circumstantial as progesterone has been proved only to inhibit gastrointestinal smooth muscle contractility in vitro.[16]

The theorized decrease in motility alone cannot completely account for the increased bile volume noted in pregnancy. Analysis of sequential films for gallbladder volume revealed a significant increase in residual volume after gallbladder emptying.[17] There is also a 50% increase in the bile acid pool associated with a concomitant steroid-induced increase in cholesterol secretion during pregnancy. Together with the replacement of deoxycholic and chenodeoxycholic bile acids with choleic acid, increased volume and decreased motility of the gallbladder enhance the development of biliary stones. When these pregnancy-induced changes occur with a metabolic and/or hereditary predisposition to developing gallstones, the likelihood of biliary disease is predictably high.

### MICROBIOLOGY OF BILIARY DISEASE

Identifiable gallstones do not have to be present in order to develop symptomatology. In fact, biliary sludge can precipitate biliary pain, acute cholecystitis, or even pancreatitis. When sludge develops into gallstones and they obstruct the cystic or common bile ducts, bacterial invasion *can* occur. However, the primary cause of acute cholecystitis is not bacterial invasion. Instead, it is frequently a chemical inflammation. After obstruction, inflammation, hyperemia, and edema of the gallbladder follow. Subsequent venous and lymphatic obstruction yields ischemia. It is only after this secondary ischemia that bacterial invasion and infection commonly occur.[18]

The presence of organisms in the bile, per se, does not necessarily imply an active infection. Bactibilia has only been reported in up to 65% of women with acute cholecystitis. In fact, most reports state that only 50% of patients with acute cholecystitis will have positive bile cultures.[19] Conversely, bactibilia is found in 20–30% of patients with biliary concrements that have minimal or no signs of obstruction. Therefore, the mere presence of biliary calculi seems to support some bacterial growth.[18,19] Ultimately, then, acute cholecystitis can be thought of as a heterogeneous disease.

The incidence of bactibilia varies significantly with the stage of disease and the patient's age. Also, as expected, with infections originating in the gut, the array of organisms in acute cholecystitis is broad. It includes both aerobes and anaerobes with a predominance of enteric gram-negative organisms (Table 1). *Escherichia coli* is the most common organism found in all studies of men and women diagnosed with acute cholecystitis. Klebsiella, Proteus, Enterococci, and Streptococci together with

TABLE 1. Common causes of cholecystitis[18]

| |
|---|
| Aerobic |
| *Escherichia coli* |
| *Klebsiella* sp. |
| Enterococci |
| *Proteus* sp. |
| Anaerobic |
| *Bacteroides* sp. |
| *Clostridium* sp. |
| Anaerobic cocci |

*E. coli* account for 75% of all bacterial strains recovered during acute cholecystitis.[18] There has been a slow increase in anaerobic isolates which is most likely due to improved culturing techniques. Nevertheless, of the anaerobes recovered, Bacteroides and Clostridia are among the most common.[18]

Although the bacteria of acute cholecystitis are undoubtedly enteric in origin, the route of entry into the biliary tract remains uncertain. Ascending infection would seem to be the most likely explanation. However, there is good evidence that hematogenous spread with direct infection through the thickened gallbladder walls also leads to acute cholecystitis.[18]

### DIAGNOSIS

To effectively treat acute cholecystitis you must first recognize the clinical picture. Other than biliary disease, the differential diagnosis in a pregnant woman with right upper quadrant pain includes preeclampsia, pyelonephritis, appendicitis, and even gastric ulcer. After initial evaluation excludes these and other less common entities from the differential diagnosis, the diagnosis of biliary disease should be further investigated. As stated earlier, differentiating cholelithiasis from acute cholecystitis can be difficult. This is especially true when pregnancy complicates the clinical picture. Some clinicians argue that differentiating between the two would not affect management. At any rate, the clinical manifestations of acute cholecystitis during pregnancy are essentially the same as the nonpregnant state.

### HISTORY

An episode of biliary pain usually begins with right upper quadrant or mid-epigastric pain which may increase in severity. Biliary colic begins quite suddenly and may radiate to the interscapular area, to the angle of the right scapula, or to the right shoulder. It may be precipitated by a fatty meal or by consumption of a large meal following a period of fasting. Typically though, biliary colic occurs in a diurnal pattern with pain peaking around midnight. Nausea and vomiting also frequently accompany episodes of colic. Finally, 70–80% of patients will have a history of colic attacks and fatty food intolerance.[13] Increasing severity of colicky pain, fever, or chills (rigors) usually implies an underlying complication, i.e., cholecystitis, pancreatitis, or cholangitis.

### PHYSICAL EXAMINATION

Physical examination reveals right upper quadrant tenderness. This is usually associated with an increase in pain to palpation upon deep inspiration (Murphy's sign). Fever and tachycardia may indicate an underlying infection. The presence of peritoneal signs, guarding, rigidity, and rebound, are extremely ominous signs and may reflect rupture of the gallbladder.

### LABORATORY FINDINGS

Laboratory evaluation may provide assistance in diagnosis but it is clear that the clinical course will dictate the management plan. Laboratory studies commonly performed when evaluating these women include blood leukocyte count, evaluation of hepatic function, serum bilirubin, amylase, lipase, and alkaline phosphatase. There are numerous alterations in several of these laboratory parameters which normally occur in pregnancy and can limit their usefulness. The blood leukocyte count varies considerably during normal pregnancy. Values can range from 5,000 to 12,000/ml and these values may rise significantly with exercise or labor. Also, alkaline phosphatase activity nearly doubles during normal pregnancy. Finally, postprandial plasma levels of total bile acids progressively increase during pregnancy. Therefore, laboratory evaluation will only provide clues in the diagnosis of biliary tract disease during pregnancy. For example, serum bilirubin may be mildly elevated and leukocytosis may be present. If bilirubin remains elevated with persistent jaundice, common duct stones should be suspected. If there is significant leukocytosis in addition, an underlying infectious process should be considered. Finally, serum amylase, lipase, and alkaline phosphatase may be of some assistance in identifying pancreatic involvement.[17]

### RADIOGRAPHIC EVALUATION

Ultrasound has revolutionized the accuracy and safety of diagnosing biliary tract disease. It has all but replaced oral cholecystography and intravenous cholangiography as the standard for diagnosis of gallbladder disease. Ultrasonography, as a rule though, provides no direct evidence of acute cholecystitis. Conversely, intravenous cholecystography gives a more functional diagnostic view of cholecystitis by directly revealing blockage of the cystic duct and biliary stasis.

Additional sonographic findings may aid in differentiating cholelithiasis from acute cholecystitis. These include gallbladder wall thickening, gallbladder distention, and tenderness upon deformation of the gallbladder with the ultrasound transducer (sonographic Murphy's sign). However, none of these findings is an absolute indicator of acute cholecystitis. In fact, when reviewing the literature, several studies both support and refute the use of gallbladder wall thickening and sonographic Murphy's sign to differentiate biliary colic from acute calculous or acalculos cholecystitis.[5,19,20]

In other words, ultrasound is imperfect in demonstrating biliary stones impacted in the cystic duct and this, unfortunately, is the cardinal sign of acute cholecystitis.[20] Nevertheless, ultrasound has no known medical contraindications or adverse side effects and should be used as the first line of diagnostic study. One study reported a 96% sensitivity and 88% specificity for ultrasound in the diagnosis of cholecystitis when combined with all of the clinical criteria previously described.[20] The overall accuracy of ultrasound in diagnosing acute cholecystitis is 93%. The same study cited above reported an overall accuracy of intravenous cholecystography as 88%; however, this was felt to be due to impaired hepatic excretion of contrast medium.

Finally, the use of intravenous contrast media always runs the risk of hypersensitivity reactions. It is, therefore, reasonable to conclude that ultrasound is accurate, safe, and the most practical method for diagnosing acute cholecystitis in pregnancy.

### TREATMENT

### Medical Management

The primary issue in the management of acute cholecystitis is when to intervene surgically. Initial medical therapy is appropriate in all patients with biliary tract disease, however, clinicians should not procrastinate when surgery is indicated. Medical therapy includes eliminating oral intake, nasogastric suction, correction of electrolyte abnormalities, and analgesia. Morphine analgesia and its derivatives should be avoided, as they may cause spasm of the sphincter of Oddi. This spasm may exacerbate an already painful condition. Finally, intravenous antibiotic therapy is usually indicated in patients with suspected acute cholecystitis, even though superinfection of the bile/gallbladder may not occur until the latter stages of disease.

The combination of a penicillin and an aminoglycoside has long been recommended as the initial treatment of choice for patients with infections of the biliary tract. This initial antibiotic therapy is empirically based on the bacterial flora likely to be encountered in the biliary tract. Aminoglycosides are used to treat gram-negative facultative biliary pathogens including pseudomonas. Penicillins offer adequate gram-positive coverage. Specifically, ampicillin will cover most anaerobes and enterococcus species. Therefore, their combination would empirically provide good antibiotic coverage in patients with evidence of acute cholecystitis.

Concern about aminoglycoside-induced nephrotoxicity in patients with jaundice and sepsis coupled with the development of newer, more effective cephalosporins has challenged penicillin plus aminoglycoside use as the preferred treatment in acute cholecystitis. Specifically, improved coverage against *Bacteroides* sp. by some second and third generation cephalosporins has led to their increased use in both prophylaxis and treatment regimes. In one study, cefepime, a third generation cephalosporin, was compared to mezlocillin and gentamicin therapy in patients with acute cholecystitis undergoing cholecystectomy. The results indicated that single drug therapy with cefepime was as effective as combination therapy with mezlocillin and gentamicin.[21] Cefepime, like many later generation cephalosporins, requires 12 h dosing and can achieve high bile, blood, and gallbladder levels. Ultimately, with increased cost consciousness, decreased drug monitoring and fewer doses mean lower overall costs related to cephalosporin use.

Piperacillin, an extended spectrum penicillin, has broad coverage against all organisms commonly bound in the biliary tract, excellent biliary excretion, and negligible nephrotoxicity. It has also been shown to be efficacious in patients with acute cholecystitis.[22,23] The overall cure rate for acute cholecystitis in patients treated with piperacillin, ampicillin and tobramycin, or cefoperazone was 92% with no difference in any of the three groups.[22] Hence, when considering antibiotic therapy in patients with acute cholecystitis, the standard of a penicillin and an aminoglycoside remains an effective regimen. However, when considering more current issues such as cost containment, alternatives including extended spectrum penicillins or third generation cephalosporins cannot be ignored.

In managing acute cholecystitis in pregnancy, many physicians advocate initial non-operative management in an effort to prevent surgical intervention prior to completion of the pregnancy. The pregnancy outcomes are highly dependent on the trimester during which the biliary symptoms occur. One study evaluated 26 pregnant patients with acute cholecystitis in all three trimesters. Only 2 of the 7 patients who presented in the first trimester had pregnancies which went to term, whereas all patients who presented in the third trimester completed a successful pregnancy.[24] Twenty of the patients did not require operation before resolution of the pregnancy. Non-operative therapy has classically been the primary goal for pregnant women with acute cholecystitis.

Having said that, there is evidence to suggest a high rate of recurrence of biliary pain in pregnancy complicated by cholelithiasis and cholecystitis. In a recent retrospective review of 44 women with biliary colic or cholecystitis, experience with conservative, non-operative therapy was not favorable. Twenty-six of the 44 patients were managed conservatively and 58% of those had recurrent symptoms. Twenty-seven percent of these women were rehospitalized two or more times during their pregnancy. The average hospital stay for these women was 14 days as opposed to 6 days for the group with surgical intervention.[25]

### Surgical Management

The implication is that cholecystectomy should be considered early in the management of these women. With regard to non-obstetric surgical therapy, several comments can be made. During pregnancy appendicitis is the most common indication for non-obstetric surgery followed by adnexal masses and cholecystectomy.[26] Cholecystectomy is performed at a rate of 1–6/10,000 pregnancies.[27] In a review of approximately 50,000 pregnancies, the preterm delivery rate within 2 weeks of any surgery was significantly higher in the third trimester than in the second trimester (25.7% vs. 8.2%). Also, there was a trend toward preterm delivery for those procedures performed emergently vs. those that were planned. Finally, in this study, tocolytic agents were not found to be beneficial.[27]

Based on the current literature, the following overall recommendations can be made with regard to management of pregnant women with acute cholecystitis. First, prepregnancy cholecystectomy is advised in those women with a history of cholelithiasis. Second, biliary colic or acute cholecystitis occurring during the first trimester of pregnancy should initially be treated medically. If possible, elective surgery should be scheduled for the second trimester. Biliary symptoms occurring in the second trimester should ideally be treated surgically. Finally, symptoms of biliary colic or evidence of acute cholecystitis in the third trimester should be treated medically, when possible. Postpartum surgical therapy should then be pursued.[25] In conclusion, due to high recurrence rates of biliary symptoms in pregnant women with acute cholecystitis, surgery should be considered early in management as opposed to postponing definitive therapy.

Once the decision for surgical intervention has been made, the question is which approach should be used. The options include endoscopic retrograde cholangiopancreatography (ERCP) with sphincterotomy and stone removal, laparoscopic cholecystectomy, or open cholecystectomy. Prior to any of the above procedures, however, appropriate antibiotics should be instituted.

With respect to ERCP with sphincterotomy and stone removal, conventional wisdom is that due to potential radiation-induced damage to the fetus, fluoroscopic procedures should be avoided. Several studies in the second and third trimesters, however, have reported successful pregnancy outcomes following ERCP with biliary stone removal.[28,29] Additionally, Hoffman and Cunningham[30] reported four pregnant women who underwent ERCP during the first trimester. In this report, radiation exposure after appropriate abdominal shielding was calculated and found to be well below levels at which damage to the fetus can occur.[30] Ultimately, then, ERCP with sphincterotomy and stone removal and possible stenting of the gallbladder appears to be a safe procedure in pregnant women with uncomplicated cholelithiasis.[31]

In cases of acute cholecystitis or complicated cholelithiasis, depending on gestational age at diagnosis, the surgical management may differ. Laparoscopic cholecystectomy in pregnancy was first reported in 1991.[32] The laparoscopic approach theoretically avoids potential postoperative complications such as thrombophlebotic and embolic events in the pregnant, hypercoagulable state. This report described a pregnancy in the early second trimester with the uterus at the pelvic brim. The patient was discharged on postoperative day 1 with an eventual successful pregnancy outcome.[32] Laparoscopic cholecystectomy has been performed successfully in non-pregnant patients with acute cholecystitis, common duct stones, previous surgical history, obesity, coagulopathy, and dilated bowel.[33] Also, gynecologic laparoscopies are frequently performed during pregnancy.[32] Therefore, a laparoscopic cholecystectomy is a reasonable consideration in pregnant women. Despite longer surgery times, reduced morbidity and mortality along with decreased cost of hospitalization justifies its use in experienced hands. Pregnancy should not be considered an absolute contraindication to the laparoscopic approach in these patients.

There is no consensus, however, at what gestational age/uterine size laparoscopy would be contraindicated. Clear contraindications such as sepsis, cholangitis, common duct stones, and portal hypertension would still apply.[33,34] If it is the opinion of the experienced surgeon that he or she cannot safely place the trocar into the peritoneal cavity or perform an open laparoscopy, the traditional laparotomy approach should be the treatment of choice. As stated previously, tocolytic therapy has been shown to be of no benefit in these patients.[26]

### CONCLUSION

Biliary tract disease in pregnancy is a relatively uncommon occurrence. Because of the heterogeneous nature of the disease, progression to acute cholecystitis can be difficult to recognize. Once diagnosed, the initial management plan should be conservative and include institution of antibiotic therapy. Then, depending on the gestational age at diagnosis, the management plan may differ. Surgical intervention, when indicated, should not be delayed and a planned intervention during the second trimester appears to offer a better outcome than surgery performed under emergent conditions.[26]

### REFERENCES

1. Birnholz JC: Population survey: Ultrasound cholecystography. Gastrointest Radio 7:165, 1982.
2. Glenn F, McSherry CK: Gallstones and pregnancy among 300 young women treated with cholecystectomy. Surg Gynecol Obstet 127:1067–1072, 1968.
3. Crichlow RW, Seltzer MH, Sannetha PJ: Cholecystitis in adolescents. Digest Dis 17:68–72, 1972.
4. Scragg RKR, McMichael AJ, Seamark RF: Oral contraceptives, pregnancy and endogenous oestrogen in gallstone disease: A case control study. Br Med J 288:1795–1799, 1984.
5. Buiumsohn A, Albu E, Gerst PH, Subbarao MJ: Cholelithiasis and teenage mothers. J Adol Health Care 11: 339–342, 1990.
6. Barbara L, Samra C, Labate AMM, Taroni F, Rusticali AG, Festi D, et al.: A population study on the prevalence of gallstone disease. The Sirmione Study. Hepatology 7:913–917, 1987.
7. Swisher SG, Schmit PJ, Hunt KK, Hiyama DT, Bennion RS, Swisher EM, Thompson JE: Biliary disease during pregnancy. Am J Surg 168:576–579, 1994.
8. Stauffer RA, Adams A, Wygal J, Lavery JP: Gallbladder disease in pregnancy. Am J Obstet Gynecol 144:661–665, 1982.
9. Chesson RR, Gallup DG, Gibbs RL, Jones BE, Thomas B: Ultrasonographic diagnosis of asyptomatic cholelithiasis in pregnancy. J Reprod Med 30:920–922, 1985.
10. Basso L, McCollum PT, Darling MR, Tecchi A, Tanner WA: A study of cholelithiasis during pregnancy and its relationship with age, parity, menarche, breast-feeding, dysmenorrhea, oral contraception, and a maternal history of cholelithiasis. Surg Gynecol Obstet 175:41–46, 1992.
11. Grundy SM, Kalser SC: Highlights of the meeting on prevention of gallstones. Hepatology 7:946–951, 1987.
12. Valdivieso V, Covarrubias C, Siegel F, Cruz F: Pregnancy and cholelithiasis: Pathogenesis and natural course of gallstones diagnosed in early puerperium. Hepatology 17:1–4, 1993.

13. Friley MD, Douglas G: Acute cholecystitis in pregnancy and the puerperium. Am Surg 38:314–317, 1972.
14. Landers D, Carmona R, Crombleholme W, Lim R: Acute cholecystitis in pregnancy. Obstet Gynecol 69: 131–133, 1987.
15. Carey MC, Cahalane MJ: Whither biliary sludge? Gastroenterology 95:508–523, 1988.
16. Fischer RS, Roberts GS, Grabowski CJ, Cohen S: Inhibition of lower esophageal sphincter circular muscle by female sex hormones. Am J Physiol 234:E243–247, 1978.
17. Braverman DZ, Johnson ML, Kern F Jr: Effects of pregnancy and contraception steroids on gallbladder function. N Engl J Med 302:362–364, 1980.
18. Bjorvatn B: Cholecystitis—etiology and treatment—microbiological aspects. Scand J Gastroenterol 90(S):65–70, 1984.
19. Thompson JE Jr, Bennion RS, Doty JE, Muller EL, Pitt HA: Predictive factors for bactibilia in acute cholecystitis. Arch Surg 125:261–264, 1990.
20. Norrby S, Frank M, Sjodahl R: Intravenous cholecystography and ultrasonography in the diagnosis of acute cholecystitis. A prospective comparative study. Acta Chir Scand 151:255–259, 1985.
21. Yellin AE, Berne TV, Appleman MD, et al.: A randomized study of cefepime versus the combination of gentamicin and mezlocillin as an adjunct to surgical treatment in patients with acute cholecystitis. Surg Gynecol Obstet 177(S):23–29, 1993.
22. Muller EL, Pitt HA, Thompson JE Jr, Doty JE, Mann LL, Manchester B: Antibiotics in infections of the biliary tract. Surg Gynecol Obstet 165:285–292, 1987.
23. Thompson JE Jr, Pitt HA, Doty JE, Coleman J, Irving C: Broad spectrum penicillin as an adequate therapy for acute cholangitis. Surg Gynecol Obstet 171:275–282, 1990.
24. Hiatt JR, Hiatt JC, Williams RA, Klein SR: Biliary disease in pregnancy: Strategy for surgical management. Am J Surg 151:263–265, 1986.
25. Dixon NP, Faddis DM, Silberman H: Aggressive management of cholecystitis during pregnancy. Am J Surg 154:292–294, 1987.
26. Kort B, Katz VL, Watson WJ: The effect of nonobstetric operation during pregnancy. Surg Gynecol Obstet 177: 371–376, 1993.
27. Morrell DG, Mullins JR, Harrison PB: Laparoscopic cholecystectomy during pregnancy in symptomatic patients. Surgery 112:856–859, 1992.
28. Baillie J, Cairns SR, Putnam WS, Cotton PB: Endoscopic management of cholelithiasis during pregnancy. Surg Gynecol Obstet 171:1–4, 1990.
29. Beck G, Brodmekel G: Choledocholithiasis in pregnancy: Treatment by endoscopic splenectomy. Gastrointest Endosc 100:A252, 1991.
30. Hoffman BJ, Cunningham JT: Radiation exposure to the pregnant patient during ERCP. Gastroenterology 38:A253, 1992.
31. Golschmiedt M, Wolf L, Shires T: Treatment of symptomatic cholelithiasis during pregnancy. Gastrointest Endosc 39:812–814, 1993.
32. Weber AM, Bloom P, Allan TR, Curry SL: Laparoscopic cholecystectomy during pregnancy. Obstet Gynecol 78: 958–959, 1991.
33. Elerding SC: Laparoscopic cholecystectomy in pregnancy. Am J Surg 165:625–627, 1993.
34. Rusher AH, Fields B, Henson K: Laparoscopic cholecystectomy in pregnancy: Contraindicated or indicated? J Arkansas Med Soc 89:383–384, 1993.
